# Report on Dental Progress

**Published:** 1855-04

**Authors:** A. M. Leslie


[To be continued.]

### ARTICLE VI.

*Report on Dental Progress.* Prepared by A. M. Leslie, D. D. S.

I. *Crystallized Gold.*—The preservation of the natural teeth does, and ever will, command the attention of the intelligent and conscientious operator. It is also rapidly gaining the attention of the more intelligent members of society in general. Hence whatever promises improvement in this direction, is of the first importance.

For a few years back, the attention of the profession has been gradually drawn to the consideration of new methods of preparing gold for plugging teeth hoped to be superior to the old mode of beating into leaf. England is entitled to the laurels for having taken the initiatory steps, but Columbia is likely to bear off the palm of victory, if victory there be on this field. While in England, Dr. Dwinelle tested some sponge gold made by Mr. Barling, of Maidstone, Kent, for Mr. John Tomes, of London, of it he speaks highly, (see Journal, vol. iv, p. 349.) His only objection, its lack of plasticity. In this same paper we are also informed that Dr. A. J. Watts, of Utica, N. Y., without a knowledge of Mr. Barling's efforts and success had produced three articles of sponge or minutely divided gold, "the first of a crystalline structure, the second possessed a laminated structure and the third a spongy arborescent form." Since the introduction of these preparations an intensity of observation has been directed to this field, which has given to it a pre-eminence in periodical discussions during the past year as well as that preceding. To pursue the history further, we find that nearly cotemperaneous with Watts of Utica, Messrs. White, of the same place, made and sold prepared or sponge gold. This was soon followed by the crystallized gold of Drs. Taft & Watts, of Xenia, Ohio. The *manner of using* which, it will be recollected was very clearly described by Dr. Taft at the last meeting of this society, both orally and in pamphlet. A curiosity was at that time expressed as to their mode of manufacture in order that if they had discovered another or improved mode of forming gold crystals, the profession might enjoy the benefit of such knowledge. It was then intimated that they were indebted to Turner and other chemists for the ground work of their process, but until experiments then in progress were completed the publication of present results would be deferred.

The writer of this report had hoped to have received valuable aid from his colaborer in this committee, Dr. Watts, in developing this branch of the report, but as yet the doctor has nothing to present on this point. Your committee is under obligations to Professor J. Taft for the readiness he has shown to communicate freely on this subject. Many of their experiments have resulted differently from what they had hoped, and the certain results are small in number, yet one thing they have discovered is of importance; this is the circumstances which control the crystallization of the gold. Instead of the size of the crystal and density of the sponge being as they, with others, thought to depend upon the relative amount of mercury used in the amalgam and the period during which it was combined, they find it to depend upon the galvanic action produced at the time the mercury is acted upon by the nitric acid; the more rapid and violent this action is, the larger are the crystals. So certain, says Dr. Taft, are their results in this respect, that they have no difficulty in making any of their specific numbers. He also says, a workable article can be made by precipitating a solution of gold with the protosulphate of iron, various degrees of sponginess being obtained by varying the rapidity of deposition. He, however, prefers crystal gold made by amalgamation with mercury and dissolving out the latter metal with nitric acid, a mode of conducting which may be found on page 257, vol. 7, Den. Recorder.

The doctor, we believe, is of the opinion that the gold now made by Watts & Co., of Utica, is prepared by a different process from that patented, whether it works differently, compared with early productions we leave others to decide.

What the process of Messrs. White, also of Utica, is, your committee cannot say, the same may also be said of the English sponge gold.

In entering upon an examination of the value of crystal gold, some have very properly inquired into its purity. This may vary with every lot; it however *must* depend on the degree of knowledge and care brought to bear by the maker. Regarding the purity of some specimens, some experiments have been made and published. Dr. E. Townsend gives on page 173, vol. 7, Den. Recorder, the results of a test made by the assayer at the Mint in Philadelphia. In 1000 parts sponge gold there were 993½ pure gold. In this respect, he says, being equal to foil in general, and superior to most. On page 37, vol. 5, of Journal of Den. Science, Dr. W. Kendal says, that some alloy of the baser metals was found in specimens subjected to analysis, and in this way he would account for the crepitation observed in some specimens when the cake is bent. Dr. Dwinelle, in an article to be found at page 346, vol. 4, of Journal Den. Science, says, "to ensure with uniformity the characteristics of adhesiveness and toughness, it is *absolutely* essential that the gold be first made pure.

"For the present it is sufficient to remark, that much of the gold prepared for our profession is rendered chemically pure by *quartation*, and consequently cannot always be pure, for the reason that gold often contains foreign metals not acted upon by acids. In addition to this, gold is often alloyed before it is beaten into foil.

"*Indicative of this fact*, and as a universal test of the quality of gold, whether in the form of foil or otherwise, it is only necessary to consider that *pure gold is of a uniform color through-*

*out the world.* A comparison of Abbey's foil, and some few others, with the paler foils of the market, will render it easy to distinguish between gold which is absolutely pure and that which is not.

"At some future time, we will endeavor to give in the Journal, the process by which gold may be obtained in all cases absolutely pure."

Dr. Taft, on page 116, vol. 7, Den. Register, says, "this preparation is pure annealed gold crystals. The method of its preparation precludes the *possibility* of its containing an alloy."

Again on page 114, he says, "there are some difficulties, however, that seem necessarially connected with the use of gold in this form (in foil.) The surface of the leaves are almost as smooth as if they were separately planished, and hence have *no facility for welding* or uniting together, so that two leaves being pressed together, only remains so because they are thus retained by the force about them. Foil that has been *perfectly* annealed after being hammered, when brought in contact under great pressure, *may* adhere *slightly*. *This inability to unite, constitutes one of the strongest objections to the use of foil.* The practice of introducing foil fillings in block, *counteracts this* difficulty as near as it is possible. Yet, fillings when thus put in, will sometimes lose small portions, and when a filling *begins* to yield, it is very soon lost; 'failing by piece-meals;' 'coming out in fragments;' 'tumbling to pieces;' &c., are remarks familiar to the ear of every dentist. A few fortunate individuals there are, however, who never have such things happen in their practice, because they have a peculiar method of *their own* for performing this operation. Of course their own method is so much better than that of any one else—always succeeding most perfectly in every operation, just because it is done in their way. It is true, that from five to twenty per cent. of all the teeth filled by reputable dentists, are not successful in the first operation. And why? Sometimes because the filling was not consolidated, or because the cavity was not properly formed to retain a filling that presented a smooth surface to its hard smooth walls, and although it was consolidated perfectly as possible, gave way either in a mass, or as is frequently the case, in pieces. In regard to this point, facts will warrant the assertion, that nine-tenths of the fillings that give way, come out in pieces; not because they were not consolidated, but because they were not, and could not be, united together into a solid mass. Let us imagine how greatly the probabilities of success would be increased, could we obtain some method of uniting the leaves of foil, that compose a filling, into a solid mass, that could not be separated even when removed from the cavity; then success would be far more certain, notwithstanding the smooth surface of the foil is presented to the walls of the cavity. The best foil fillings that are put in by the most superior operators, when removed from the cavities, can be torn to pieces by very slight efforts, and a great many even fall to pieces before they come out of the cavities."

Speaking of crystal gold, he says, "The fitness of this preparation of gold, for filling teeth, is quite apparent when we consider all its properties. It is of such a consistence, that it can, by compression be perfectly adapted to a cavity of any form. The part of the filling presented to the walls of the cavity, is not, as with foil, a smooth, but crystallized surface; the points and edges of the crystals are presented to the walls, and take hold upon them.

"It is preferable to use as large pieces as is admissible, in any given cavity; for a large piece can be just as easily and perfectly condensed, as a small one; quite the contrary is true in regard to foil. A much more solid filling is obtained with this preparation than with foil. In proof of this, we only refer to the fact, that a filling of it is about one-fourth heavier than the same sized filling of foil. But then, upon this an objection is raised, 'that it requires more gold to fill a tooth;' so it does. I do not now intend to answer this objection, but only remark, that the more gold that can be put into a cavity of a given size, the better for the patient; and in nine cases out of ten, they will not refuse to pay for it."

In Dr. Townsend's article, before quoted, we find an experiment of his showed the crystal gold filling to be one-fifth heavier; also, that when two approximal surfaces were filled and met at the neck the gold had become "as hard as iron or even harder."

Dr. Dwinelle says, "as a test of density, well formed plugs do not shrink under the blow-pipe, their inner surfaces are bright and solid, while their polished disks take the graver like plate."

Dr. Ballard, in Den. News Letter, vol. 7, page 155, says, "the same force will with crystal gold make a harder and more solid filling than if gold foil were used." He sums up its advantages thus, "1st, a saving of labor; 2d, of time; 3d, a saving of tooth substance, it needing less under cutting in shallow cavities; 4th, better operation when completed."

Of the gold prepared by Watts, of Utica, he says, "I am satisfied it will prove in *every case* better than the best foil."

Dr. Townsend says, in the article before noticed: "I do not think sufficient time has elapsed to prove incontestably its usefulness, or its superiority in those cases where the difficulty of using foil is considered insurmountable; nor do I think it will ever supersede the use of foil as a permanent filling."

Dr. Dwinelle writes: "Although we consider Dr. Watt's sponge gold indispensable to our practice, yet we do not think it will ever entirely supersede the use of gold foil: It can often be used with great advantage in combination with gold foil."

Prof. A. A. Blandy, in a paper on sponge gold, says: "A more extended experience than any one has yet had (April, 1854,) with crystallized gold is necessary to an absolutely correct judgment with regard to its relative value as compared with foil, but from the experiments I have made, I am disposed to believe it will prove an invaluable acquisition to dental practice. Indeed, I almost feel warranted in the assertion, that with an equal amount of labor better fillings can be made with it in the grinding sufaces of molar and bicuspid teeth, and in large cavities easy of access in approximal surfaces of most teeth than with foil. But in small cavities, and especially in the approximal surfaces of teeth, not easily reached, and partially covered by the edge of the gum, foil can certainly be used with much greater facility and certainty."

Professor J. D. White says, in News Letter, July, 1854, of sponge gold: "When it is put into a cavity in mass it cannot be made hard or dense to any distance below the surface, and if it is used in small particles it requires too long a time to fill a cavity, and as it requires so long a time, the mouth, as a general thing, becomes too wet to allow the plug to be finished, and without great care it cannot be made to adhere again, so as to continue the completion of the plug, and if the plug cannot be made much more solid than a foil plug, it will not remain in the cavity as well. The dampness of the mouth or the tooth will break up the *texture* of the plug, and cause it to crumble out. We have had this to occur with us in many instances, and it has also occurred with those who are much in favor of its use. An ordinary foil plug is much better than a *good* sponge plug.

Dr. Blandy says, that the effect of *moisture* on the surface of a plug during filling makes it almost impossible to add a thin layer if needed.

Dr. Townsend found water to change the properties of sponge gold.

Professor White says, one piece rolled a little in the fingers will not adhere to another piece.

Dr. A. J. Watts, in his directions, says, respecting moisture: "In case the gold should become wet during the operation of filling, thoroughly condense the gold already in the cavity, burnish its surface, and let the patient rest;" before proceeding further, you are to slope the surface of the stopping. "In case the gold loses its adhesive quality by *dampness* or exposure, *dry* it over a spirit lamp."

To remove *moisture* and restore its *adhesive* qualities, Dr. Taft recommends in the January, 1855, number of the Register, that the gold be annealed. Mr. Kendall, in the January No. of the A. Journal, also recommends heating before using to remove the moisture absorbed from the atmosphere.

In these quotations we have endeavored to present you with the advantages claimed for sponge or crystal gold over gold foil, also to present you as succinctly as possible, under the hurry in which this report was thrown together, the experience of those who highly approve it, and some who are not so fully wedded to its use. With a review of a few points gone over, we leave the further discussion of the subject to the society.

The writers on this subject have as a matter of course taken gold foil as the standard by which to measure the merits and demerits of sponge and crystal gold, but unfortunately for some of their conclusions, they have not all been possessed of correct knowledge on the properties of gold foil, or the method of its manufacture—hence the inferences drawn therefrom, (and some of them were very important,) in favor of crystal gold, being erroneous, gold foil may not yet be compelled to live in the shadow of this precocious growth.

There is nothing perhaps more contagious than the cry of impure! impure!! Give a dog a bad name, and ten to one but he has had his day. Many a dog has received a "metallic" stopping who deserved better doctoring from his faithfully served friends.

Dr. Dwinelle says, much of the gold (that is, foil,) prepared for the profession is rendered "chemically pure by quartation, and *consequently* cannot always be pure, for the reason, that gold often contains foreign metals *not acted upon by acids!* Indeed; then how does the doctor propose to purify it, without acids? by cuppelation, cementation, or how? Let us pray the doctor to hurry up that article he promised "at some future day," in which he is to develop "the process by which gold may be obtained *in all cases absolutely pure*, and give us the agent or agents which will remove the foreign metals found in gold more perfectly than now may be done by acids.

For want of chemical knowledge great ignorance prevails respecting the purity of gold foil. The writer of this has yet to meet the manufacturer of foil whom he believes foolish enough "to *alloy* gold intended for foil before beating it." He believes the makers universally do their best to produce a *pure* article, and further, that they very generally succeed; but it is with some gold beaters as it is with others, they lack the practical chemical knowledge demanded by their professional wants, and an imperfect article is the consequence.

Undoubtedly Dr. Dwinelle is right when he says, "*pure gold is of a uniform color throughout the world;*" but he falls into an error in which he will find abundance of company when he gives Abbey's foil *as a standard of color* to test pure gold by throughout the world.

This can be shown in various ways, 1st. Abbey's foil used to be, and we suppose yet is, occasionally *variable in color.* 2d. That peculiar red color it possesses is only superficial; melt a leaf or two of it with the blow-pipe, and you will find it range in color with some of "the paler foils of the market." It wears a livery, which has been taken by many beside the doctor to indicate its royalty. Some thirteen years ago the firm of A. & J. Lester, of Cincinnati, made an exactly similar article, and sold it extensively in the west for several years. They were the first to make an article similar to Abbey's foil. Others beside Messrs. Abbey were making it in the west a few years ago, and may still be.

What this coloring matter is may be difficult to determine, but of one thing be assured—*it is only present when iron is made use of in the process of purification,* and need not be then if the gold is *perfectly washed;* take it then, not in the sponge, but melt it, and you have a color I am willing should be taken as a standard for "all the world" to estimate its purity of foil by. So much for the impurity of foil. A few words on the purity of sponge or crystal gold. It will never do to estimate its purity by the color, for various reasons: 1st. The texture varied by rapid or slow crystallization, or in sponge by rapid or slow precipitation, will vary to some extent the effect of light reflected from it to the eye. 2d. The *degree of light* to which it is subjected when annealed will vary the color, especially in sponge gold. Should you wish to bring it to the test of color, melt a portion. But there are better tests than this for purity. Trusting we have by this time relieved the consciences of some of those who have done much plugging with foil, we will take up the next, in which it is decried, viz. its want of adhesiveness. This is said by Dr. Taft to "constitute one of the strongest objections to the use of foil. The advantages of the new preparations over foil in this respect has been by other writers less forcibly expressed.

It will probably be remembered by those who were present at our last yearly meeting, that when this point was under discussion, the writer briefly alluded to the property possessed by gold of uniting firmly, or welding by simple pressure, so that in this way the holes unavoidably made in manufacturing gold leaf for gilding purposes were stopped, as it is called, by placing a piece over each hole, slightly larger, and pressing it down with the finger, and that in the gilder's work this patching does not show. He also stated, that gold foil could be made without any difficulty, which would possess the adhesive property so perfectly when annealed, that simply the weight of one leaf laid upon another would forever unite them at the points of contact.

Fifteen years ago just such foil was supplied by the firm before alluded to, to a prominent member of the profession in the west; and while he would use no other, other operators they supplied preferred another kind. He accounted for it by saying, they knew not *how* to use such as he preferred. Mr. Jas. Lester, the successor of A. & J. L., informs me that he has recently filled orders for foil possessing this quality. Doubtless by adopting the method of insertion required by such foil some improvement might result.

It may be presumed that every manufacturer of good foil can supply such an article. It will never be found to possess the deep color of Abbey's foil, as when that is present in sufficient quantity it *cannot be made to adhere.* Dr. Taft informs us that he recently made some experiments at the O. D. College with foil and crystal gold, in one of which he tested the adhesive quality of the foil already alluded to. The filling when removed did not fall to pieces, but bore cutting in two.

In view of all these considerations, we trust we have in part redeemed foil from the second charge—"a want of adhesiveness."

3d. It is contended that a "more solid filling is made with the new preparations than can be with foil."

According to Dr. Townsend's experiment, one-fifth more can be put in the same cavity. Dr. Taft estimates a greater amount, "about one-fourth." Dr. T. states, that in recent experiments, before alluded to, a student put in more weight of foil by the block method than he could get in the same cavity by using crystal gold. It is evident then, that the relative amount is as yet unfixed.

Dr. Ballard says, the same force will make a harder and more solid filling than foil.

Now, how is this? Is it more yielding than No. 4 gold foil? that more weight is got in the same amount of pressure, or is it the dense state of the particles *before compression* that allows of more weight being placed in a cavity? It seems hard to admit the former. If the latter is the cause, what is gained by it. In estimating fillings made by the *same material*, we may grant weight to be a very fair measure, but in comparing dissimilar preparations it is not so readily to be allowed. It might perhaps fail to approve different specimens of crystal or sponge gold. Let us then not too readily admit this as a means of condemning gold foil. The true tests of a gold or any other filling are its ability to *preserve the supporting walls of denture from the action of destructive fluids, and preserve its own surface intact.* That this is frequently done equally well with tin as with gold, shows that weight is no absolute test. It may yet be that a lighter material than either may be found for filling teeth, but we have done.

We believe we have met the main complaints against foil fairly, we hope in such a manner as exhibits the true progress made in this matter. That crystal and sponge gold may, after a similar trial to that which gold foil has endured, prove to possess advantages over the best foil, I sincerely wish. In the mean time let us not fear to use foil on the score of its impurity, nor adhesiveness, or the relative weight.

*Folding Foil.*—Richard Burnet, of Belfast, Ireland, presents the profession an instrnment worthy of notice, for folding and crimping strips of gold, so as to facilitate the doubling foil where strips are preferred. (See D. Recorder, vol. 7, page 172.)

*A New Non-Conductor.*—In the News Letter, vol. 7, page 138, C. A. D. Bonchel, M. D., D.D.S., gives us the result of various experiments. Horn, pared or filed to proper thickness, as the best non-conductor, it is softened in warm water before applying.

*Sensibility of Dentine.*—Dr. Harris in the April No. of the Journal recommends the use of chloride of zinc for removal of exalted sensibility of dentine preparatory to filling. He says: "The chloride may be applied on a little raw cotton, or lint, or it may be mixed with an equal quantity of flower, the atmosphere furnishing moisture enough for the formation of a paste, or it may be mixed with a little pure auhydrous sulphate of lime in impalpable powder, and then applied to the tooth; one application of from five to ten minutes is ordinarily enough.

Professor Taft and Dr. Watt state that they have lately made use of an ethereal solution of terchloride of gold to remove sensibility of the dentine. Its possessing a larger share of chlorine than the zinc preparation, they believe it acts more rapidly; care, they say, is needed in its use, as the gold in solution stains the part it is evaporated upon. Dr. B. T. Whitney, in the April No. of the Register, recommends nitrate of silver for this purpose preparatory to plugging; also, when caused by denudation, he contends the mouths of the cells are closed by the silver.

Professor White, in October No. of News Letter, says he has used chloride of zinc for nearly twelve years for relieving sensisibility of dentine preparatory to plugging. The general rule for its use is to apply it on cotton until the pungency it produces passes off.

Connected with the plugging of teeth we are pleased to note a very perfect little instrument presented to the profession by Dr. Arthur, viz. the Saliva Pumps. It is a very useful instrumen, when a very profuse flow of saliva occurs in plugging difficult cavities.

*New Mode of Noting Fillings. By Dr. W. S. How.*—This consists in the use of diagrams, easily made in the note-book, and is very serviceable when the use of an ambler or similar journal is not preferred.

The basis of this mode is square diagrams for the upper teeth, and circular ones for the under. The teeth of the *right* side are indicated by a short line running from the upper right hand angle. The left teeth by a line from the upper left angle by various rapidly made marks *each* tooth is indicated, and on the diagram when made he marks the filling and filing. As an algebraic expression for operations, it possesses great merit.

"*A New Method for Operating on Loose or Sensitive Teeth. By W. A. Helm.*" *(See American Journal, vol.* 5, *p.* 33.*)*—Dr. Helm proposes the use of plaster of paris around loose teeth preparatory to plugging or filing. We have in this a rediscovery, and another evidence of the need of more carefully reading the past history of our profession. Doubtless Dr. H. was ignorant of the fact that Dr. C. C. Allen had preceded him in this matter six years. On page 21, vol. 3, Den. Recorder, will be found "A New Method of Supporting Frail Teeth while Filling." "In such cases great strength may be given while packing the filling by coating the teeth with good plaster of paris." We tried Dr. Allen's suggestion years ago, and believe it serviceable. Honor to whom honor is due is a rule in every liberal profession.

